# Changes in the Epidemiology of Hepatocellular Carcinoma in Asia

**DOI:** 10.3390/cancers14184473

**Published:** 2022-09-15

**Authors:** Yao Liu, Lianxin Liu

**Affiliations:** 1 Department of Hepatobiliary Surgery, The First Affiliated Hospital of USTC, Division of Life Sciences and Medicine, University of Science and Technology of China, Hefei 230001, China; 2 Anhui Province Key Laboratory of Hepatopancreatobiliary Surgery, Hefei 230001, China; 3 Anhui Provincial Clinical Research Center for Hepatobiliary Diseases, Hefei 230001, China

**Keywords:** hepatocellular carcinoma, influencing factors, changing epidemiology, Asia

## Abstract

**Simple Summary:**

The incidence and mortality of hepatocellular carcinoma (HCC) in Asia are among the world leaders. By understanding the changes in prevalence and influencing factors of HCC, we can better understand the current situation in Asia and take measures to reduce the incidence.

**Abstract:**

Hepatocellular carcinoma (HCC) is one of the most common cancers worldwide, with high morbidity and mortality, and the incidence is on the rise. HCC imposes a heavy healthcare burden on Asian countries due to the presence of multiple HCC risk factors in this area. Chronic hepatitis B virus (HBV) infection, chronic hepatitis C virus (HCV) infection, non-alcoholic liver disease (NAFLD), aflatoxin and alcohol intake are the causes of HCC that cannot be ignored. Compared with the pre-vaccination era, universal vaccination of newborns reduces the incidence of HCC. Anti-viral therapy with nucleos(t)ide analogues also causes a decline in HCC incidence. Early screening and direct-acting antiviral agent are beneficial to the prevention and treatment of HCV. For HCC caused by NAFLD and other reasons, lifestyle changes are imperative. This paper introduces the epidemiological trends of HCC in Asia and highlight future efforts. Focusing on prevention may be the most effective way to improve the prognosis of this hard-to-treat cancer.

## 1. Introduction

Liver cancer is one of the leading causes of cancer-related death in the world. The World Health Organization estimated that in 2020 there were about 905,677 new cases of liver cancer and about 830,180 people died of liver cancer in the world [1]. Hepatocellular carcinoma (HCC) is mostly mentioned when it comes to liver cancer and causes an important living and economic burden around the world, especially in Asia [2,3].

In Asia, liver cancer is the fifth most common cancer and the second most common cause of cancer-related death [1]. In 2020, there were about 609,596 new cases of liver cancer in Asia, accounting for about 72.5% of the total incidence of liver cancer in the world, with Age-Standardized Rate (ASR) per 100,000 of 11.6. As for mortality, liver cancer-related deaths in Asia were about 566,269, accounting for 72.4% of the total liver cancer deaths in the world, with ASR of 10.7 [1]. The incidence and mortality of liver cancer are higher in Asian men than in Asian women. Asian men have the fourth highest incidence of liver cancer and the second highest mortality. While for Asian women, the incidence of liver cancer ranks seventh and the mortality ranks sixth. In 2020, there were 471,999 cases of liver cancer reported in men and 184,993 in women in Asia. In terms of liver cancer deaths, 437,697 Asian men and 184,993 Asian women died of liver cancer [1].

**Figure 1:**
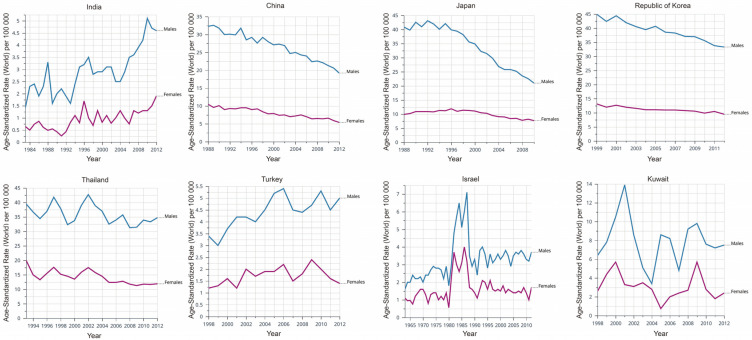
Dynamic changes of the incidence of liver cancer in different Asian countries, based on CANCER OVER TIME|IARC (International Agency for Research on Cancer). Data are presented as incidence per 100,000.

The incidence of liver cancer in Asian populations shows different trends (Figure 1). East Asian regions (e.g., South Korea, Japan and China) and Southeast Asian regions (e.g., the Philippines) have significant declines of the incidence rates of liver cancer, as reflected by average annual percent change (AAPC) [4]. A study surveyed eight countries in Asia and discovered that six of them has a declined incidence rate since 1978, including China (AAPC = −1.6%), South Korea (AAPC = −2.2%) and the Philippines (AAPC = −1.7%), but rates has leveled off in South-Western Asia (e.g., Israel) [4]. Although most liver cancers turn out to be HCC, it is worth noting that HCC only takes up about 27% of liver cancers in Thailand. Although the incidence of primary liver cancer has grown in Thailand, the incidence of HCC has declined sharply since 2000 [4]. Moreover, GLOBOCAN 2020 shows a worrying trend that the incidence of liver cancer in many countries increasing significantly in recent years, including Iran, Afghanistan, Qatar, Azerbaijan, Iraq, and Nepal. Local governments should pay attention to this rising trend to avoid a large-scale epidemic of liver cancer [5].

## 2. Viral Hepatitis-Related HCC

### 2.1. Hepatitis B Virus (HBV)

HBV activates oncogenes and suppresses anti-oncogenes by integrating the genomes of oncogenic viral proteins into the host genome, leading to the occurrence of HCC [6]. Even for patients without cirrhosis, HCC occurs frequently in chronic HBV carriers, suggesting that HBV could be carcinogenic itself [7]. Hepatitis B virus infection is moderately to highly prevalent in the Asia-Pacific region, accounting for 75% of chronic hepatitis B positive patients globally [8]. We could find the highest incidence of liver cancer in East Asian countries, accompanied by a high rate of HBV [9]. The prevalence of HBV is almost 18% in China, 5% in India and 4% in Japan [10,11]. However, HBV vaccination in neonates has significantly affected the epidemiology of chronic HBV infection, which has declined in most parts of the world from 1990 to 2005, including Asian countries [12].

In Singapore, HBV infection is accounted for most liver disease and 66.6% of liver cancer-related deaths according to the GHE 2015 dataset [13]. Luckily, this situation has changed. The incidence of liver cancer in male decreased from 27.4 cases per 100,000 population in 1973–1977 to 17.2 cases in 2008–2012, while female incidence decreased from 6.9 cases per 100,000 population in 1973–1977 to 4.8, which may be attributed to the inclusion of HBV vaccination as part of Singapore’s national childhood immunization program in 1985 [14].

In 2001–2004, among those born in 1977–1980, the mortality rate due to HCC decreased significantly between the ages of 5 and 29 years (0·81 deaths per 100,000 for carcinoma to 0·05 per 100,000) in Taiwan, thanks to the immunization campaign in 1984 [15]. Of note, although the incidence of hepatocellular carcinoma has decreased since the 1984 immunization campaign, a community-based outreach screening in Taiwan estimated a total number of around 3 million HBsAg carriers from 1996 to 2005, indicating that the number of HBV carriers is still large [16].

The incidence of HCC in all age groups in Hong Kong has declined significantly over the past 25 years, partly due to a decline in HBV infection rates since the introduction of universal HBV vaccination in 1988. However, chronic HBV infection was still the main reason causing HCC in Hong Kong between 1992 and 2006, taking up 80% of all causes of HCC in 1992 and 78% in 2006 [17].

In addition to the promotion of vaccines, using nucleos(c)ide analogs (NAs) to inhibit the virus can improve liver inflammation and reverse liver fibrosis, thereby preventing chronic hepatitis from developing into cirrhosis and eventually developing into HCC [18]. Numerous studies have demonstrated the role of NAs in reducing HBV-related HCC. A Japanese study showed that the 5-year cumulative incidence of HCC in chronic hepatitis B (CHB) patients treated with entecavir was significantly lower than that in control groups [19]. In Hong Kong, entecavir-treated patients had a significantly lower risk of developing HCC compared with untreated cirrhosis patients [20]. A study in Taiwan also confirmed the role of NA in the development of HCC. CHB patients treated with NAs had significantly lower 7-year HCC incidence compared with untreated patients [21].

### 2.2. Hepatitis C Virus (HCV)

Chronic HCV infection is also an important cause of HCC. The prevalence of HCV is 10% in Mongolia, 6.7% in Pakistan, 2.7% in Thailand, 1.3% in China and 1% in India [22]. In 2012 HCV instead of HBV has become the most common cause of HCC in Pakistan and up to 58% of cases of HCC are attributable to HCV [23]. The main causes are the reuse of syringes and medical devices, contaminated blood or blood products, and the reuse of razors [24]. Notably, mass screening, treatment of 510,000 HCV cases per year in Pakistan, effective awareness campaigns and use of sterile equipment is estimated to significantly reduce HCV burden and prevent 116,000 new liver cancers over the next 15 years [25].

From 1958 to 1970, there was no significant change in hepatitis related HCC mortality in Japan. However, the incidence and death of HCC has increased exponentially since 1970 and peaked in 21st century, most likely due to the increase in HCV infections following World War II [26]. After a plateau in 2002–2004, the number of deaths due to HCC began to decline, reaching 28,889 in 2015 [27]. Between 1981 and 2003, the HCC incidence rate in Japanese men was higher than in women. Over 22 years, the incidence of male HCC increased from 29.2/100,000 cases in 1981 to 41.9/100,000 cases in 1987, and then fluctuated within the same range for eight years until a downward trend was observed in 1995. The incidence of liver cancer in women remained stable [28]. Despite the high incidence of HCC in Japan, the etiology is different from that in other Asian countries. In Japan, chronic HCV infection is more common than HBV infection, accounting for 79% of HCC and only 11% of HCC with HBV infection [29].

In the past twenty years, India’s HCC incidence increased, especially in Mumbai, Chennai and Bangalore. In the study cohort of 213 patients with HCC from 1999 to 2005, 83.1% were men, and the incidence of HCC was higher in men than in women [30]. In India, the main causes of HCC include HBV infection, HCV infection and alcohol consumption. HBV positive patients accounted for 70.42% [31]. Despite data showing that the incidence of HCV-related HCC in Asia has been decreasing since 2006, the potential risk of HCV infection cannot be ignored [5].

## 3. NAFLD and Obesity-Related HCC

NAFLD is defined as having more than 5% fat accumulation in liver cells, with the absence of HBV or HCV infection, and no excessive alcohol consumption [32]. NAFLD is gradually becoming the most common chronic liver disease in developed countries. However, due to the westernization of dietary habits in Asian countries, NAFLD may also be prevalent in the near future [33]. NAFLD is strongly associated with HCC [34]. Data on the impact of autoimmune hepatitis, primary biliary cholangitis, primary sclerosing cholangitis, and IGG4-related liver diseases on liver-related morbidity and mortality in the Asia-Pacific region are insufficient, so the following sections will be focused on NAFLD.

The etiology of HCC in Asia is undergoing a transition from viral factors to non-viral factors, including NAFLD. According to GHE2015 data, NAFLD and other causes account for 10.5% of all liver cancer deaths in mainland China [13]. Changing lifestyles and eating habits have led to an increase in the prevalence of NAFLD in mainland China [35]. The estimated total number of epidemic NAFLD cases in China in 2016 was 243.67 million and 7000 cases became HCC [36]. The prevalence of NAFLD-associated HCC cases is estimated to be increasing in all countries/regions studied, such as 47% in Japan (from 2200 cases in 2016 to 3240 cases by 2030). Among all years, China had the highest incidence of HCC, increasing from 14,090 cases (2016) to 26,240 cases (2030), an increase of 86%, whereas Japan saw the lowest increase (44%), from 1050 cases a year to 1520 [36]. In Korea, a study found that the proportion of HCC patients associated with NAFLD increased from 3.8% in 2001–2005 to 12.2% in 2006–2010 [37]. Although data for other countries are lacking, the incidence of NAFLD-related HCC is expected to increase rapidly in the future, considering that many Asian countries have developed NAFLD in the past two decades due to sedentary lifestyles and over-nutrition [38].

As a liver manifestation of metabolic syndrome, NAFLD is closely associated with obesity. Asia is the fastest growing country in terms of obesity, with China, for example, seeing a 414% rise in the prevalence of overweight and obesity between 1982 and 2002 [39]. Several population-based studies from Taiwan have shown that metabolic syndrome significantly increases the risk of HCC in patients with HBV or HCV. Being overweight leads to increased lipid peroxidation and oxidative stress and then makes it easier for chronic hepatitis B to become liver cancer [40]. A study in 2008 found that diabetes is also an HCC susceptibility prediction index, maybe associated with HBV and HCV infection status [41]. Diabetes contributes to a higher risk of HCC progression in HCV-infected patients and a higher risk of all-cause mortality in patients with or without HCC [42]. According to several national health surveys in Asia, the prevalence of overweight and obese people has increased [43]. Epidemiological studies have shown that type 2 diabetes mellitus (DM) is also a major risk factor for liver cancer and is commonly associated with NAFLD [44]. By 2030, it is expected that the largest group of DM patients will be found in India and China. The other four Asian countries including Indonesia, Pakistan, Bangladesh and the Philippines are among the top 10 countries with the highest prevalence of DM. It is not hard to see that obesity and DM may play a role in liver cancer in the future [45].

## 4. Aflatoxin-Related HCC

Aflatoxins are a family of carcinogens produced by fungal species such as Aspergillus flavus, Aspergillus parasiticus, and Aspergillus nomius in warm, humid environments [7]. In Asia, these mycotoxins contaminate foods such as corn, peanuts and soybeans and cause liver damage [46]. Long-term low level dietary exposure to aflatoxin is a risk factor for HCC in Asia [47].

One reason for the decline in liver cancer incidence rates is the aflatoxin reduction program. Food processing plants of Philippines voluntarily began monitoring aflatoxin, leading to a significant reduction in its levels, thereby reducing the incidence of HCC [48]. Qidong once had the highest incidence of liver cancer in the world. Since 1985, policies allowing rice to be substituted for corn in the diet have resulted in a 40-fold reduction in aflatoxin-albumin adducts. This resulted in a significant decrease in the incidence of HCC in men (ASR = 89.9 from 1983 to 1987, ASR = 60.9 from 2008 to 2012, −32.3%) and a slight decrease in women (ASR = 24.5 from 1983 to 1987, ASR = 21.5 from 2008 to 2012, −12.2%) [48,49]. The proportion of aflatoxin-albumin adducts in randomly selected serum samples collected since 1980 was found to have decreased from 100% in 1982 to 23% in 2009 and 7% in 2012. It is considered that 65% of the decrease in primary liver cancer deaths in Asia is resulted from the reduction in aflatoxin exposure between 1982 and 2009 [49].

## 5. Alcoholic Liver Disease

Many studies have shown that heavy drinking (>50–70 g/d over several years) is associated with HCC [50]. A relatively low level of aldehyde dehydrogenase, mitochondria (ALDH2) among Asian populations may exacerbate liver damage caused by alcoholism [51]. Alcohol intake in Asia plays a smaller role in HCC than in the United States and Europe. In Middle East countries, alcohol consumption is relatively low [52]. Although research on alcoholic liver disease trends in Asia is limited, the incidence of alcoholic liver disease is also increasing annually among hospitalized patients [35].

The most recent data, from 2014, showed that Asia-Pacific countries’ drinking scoring patterns were divided into two main tiers: China, Japan and Singapore scored 2. In Japan, the ratio of HCC patients with non-viral causes continues to grow, and the lifelong alcohol consumption could be a potential cause [53]. The other tier includes other countries such as India, the Southern Republic, Malaysia, the Philippines, Thailand, Indonesia, Laos and Cambodia, with scores of 3 [54]. A high score is linked to a higher risk of drinking [55]. For the definition of drinking scoring patterns, please refer to Rehm et al. [56]. The increase in alcohol intake across the Asia-Pacific region between 2006 and 2016 May have contributed to an increase in age-standardized liver cancer rates [57]. The reason for the increase in alcohol consumption is not clear, but it could be due to lower taxes on alcohol [58].

## 6. Estimated Trend of Liver Cancer in Asia

**Figure 2:**
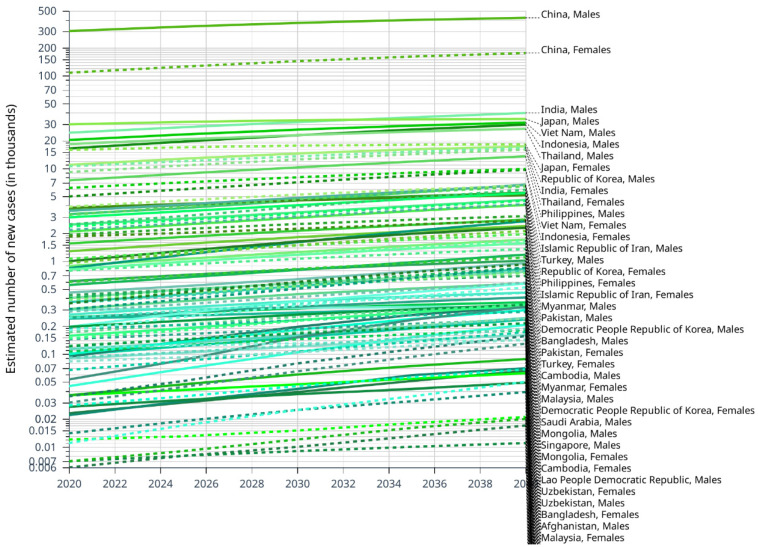
Estimated number of new cases of liver cancer from 2020 to 2040, Males and Females, age [0–85+], based on CANCER TOMORROW|IARC.

We estimated the number of the future incidence rate of liver cancer by 2040 by CANCERTOMORROW|IARC. Overall, China may have the highest total number of liver cancer cases in Asia. Until 2040, the incidence of liver cancer in male is almost always higher than in female in Asian countries (Figure 2). We speculate that this trend is inevitably related to lifestyle. Countries where the incidence of liver cancer is expected to increase rapidly include the United Arab Emirates, Qatar, Kuwait and the Syrian Arab Republic. The rise of incidence rate in Japan, Georgia, Democratic People Republic of Korea Armenia and China is relatively flat (Table 1).

## 7. Prevention Methods of HCC in Asia

### 7.1. Prevention of Viral Hepatitis-Related HCC

Most HBV infections are caused by vertical transmission, that is, mother-to-child transmission [59]. Preventing HBV infection is an important step to prevent HCC. Compared with the pre-vaccination era, universal vaccination of newborns can significantly reduce the incidence of HCC. WHO recommends that all newborns get HBV vaccine within 24 h [60]. Some countries recommend that hepatitis B immune globulin be administered at the same time as the vaccine to establish passive immunity, this combination is more effective [61]. In patients with chronic HBV carriers, certain factors can increase the risk of HCC, including advanced age, males, cirrhosis, drinking, chronic HCV or HIV infection, metabolic syndrome, and genetic polymorphisms [17,62]. Although many of these factors cannot be changed, antiviral drugs can be used to significantly control viral replication and reduce HBV DNA levels, such as interferon (IFN) and nucleoside analogs (NA) [59].

HCV-related liver cancer is another avoidable cause of primary cancer. Recent guidelines recommend screening infants and people at risk of HCV infection [59], even covering everyone born between 1945 and 1965 [63]. In addition, the AASLD/American Academy of Infectious Diseases guidelines recommend that all patients with chronic HCV infection should receive antiviral treatment [64]. For HCV, direct-acting antiviral (DAA) drugs could remarkably increase the cure rate. Therefore, all HCV patients should consider DAA treatment.

### 7.2. Maintain a Healthy Lifestyle

In a meta-analysis of 38,940 cancer cases, Esposito et al. [65] found an association between metabolic syndrome and HCC, and the association was stronger in Asians. The risk factors of NAFLD-related HCC include NAFLD, obesity, Patatin-like phospholipase-3 gene, diabetes, and metabolic syndrome [59]. Although the results of clinical trials have shown that potential treatments for NASH are inconsistent, diet and weight loss have brought positive results for NAFLD patients [66]. Weight loss of 7% to 10% is an intervention goal for most lifestyles and can improve liver enzymes and histology [59]. In addition, according to the AASLD guidelines, every NAFLD or liver cirrhosis patient should have a HCC screening every six months [67]. For patients with type 2 diabetes and metabolic syndrome, weight loss and regular aerobic exercise can reduce insulin resistance and improve inflammation [68]. In China, overweight and obesity increased by 414% between 1982 and 2002, which may contribute to NAFLD becoming an increasingly common cause of HCC in Asia [69]. Although lifestyle factors are strongly associated with NAFLD and an association with HCC has been demonstrated in animal studies, data on the relationship between humans and HCC are scarce. In animal models, the incidence of HCC in animals with exercise behavior is lower than in sedentary animals [70]. A systematic review and meta-analysis of 14 prospective studies confirmed an inverse association between physical activity and HCC risk [71]. However, due to the complex differences among individual lifestyle factors, it is difficult to accurately analyze their impact.

Several studies have shown that consuming vegetables reduces the risk of liver cancer. In the Shanghai Women’s and Men’s Health Study [72], which included 267 cases of liver cancer, vegetable-based dietary patterns were inversely associated with liver cancer, such as high intakes of celery, mushrooms, scallion vegetables, and beans/legume products. In Japan, vegetable consumption was also associated with a similar reduction in HCC risk [73]. Certain phytochemicals found in fruits, vegetables, herbs, and medicinal plants and dietary antioxidants, such as coenzyme Q, vitamin C, vitamin E and selenium, have been recommended to prevent HCC [74]. Furthermore, the Mediterranean diet also has a protective effect on HCC patients [75].

Reducing dietary aflatoxin exposure is a key step in reducing the prevalence in areas with high HCC epidemic burden [76]. Beginning in the 1980s, several agricultural policy reforms, such as improved storage of rice and maize, were implemented in highly aflatoxin-exposed areas such as eastern China, which resulted in a 65% reduction in HCC-related mortality between 1982 and 2009 [77].

A meta-analysis conducted by the World Cancer Research Foundation found that every 10 g of alcohol intake per day increases the statistical risk of HCC by 4%, avoiding heavy drinking can reduce HCC burden [78]. Increasing alcohol consumption among the Asian population plays an enormous role in the burden of HCC in this part of the world and is a matter of great concern [79].

In addition to diet and exercise factors, smoking is well known to be a risk factor for HCC, quitting smoking may of great value in preventing HCC and other malignant tumors.

### 7.3. Pharmacological Therapies

Sorafenib has been approved for the treatment of advanced HCC patients worldwide with Child-Pugh grade A liver function and produced similar survival benefits in this diverse patient population [80]. Patients with Child-Pugh grade B liver function do not have excessive risk with sorafenib, but they are more likely to develop liver decompensation. Therefore, sorafenib should be used with caution in patients with Child-Pugh score 7 and is not recommended in patients with Child-Pugh score > 7 or decompensated cirrhosis [80]. Regorafenib has stronger inhibitory activity on a variety of angiogenic pathways and carcinogenic pathways [81]. In addition to sorafenib and regorafenib, there are a variety of molecular targeted drugs, including sunitinib, brivanib, linifanib, ramucirumab, erlotinib and everolimus have also been investigated. However, they did not show a survival advantage in a phase III randomized controlled trial [80].

In recent years, significant breakthroughs have been made in agents targeting immune checkpoint proteins such as cytotoxic T-lymphocyte antigen-4 and programmed cell death-1 [82,83]. Their cancer-killing effects are achieved by maintaining T cell activation. Clinical trials of immune checkpoint inhibitors alone or in combination with immunotherapy are ongoing [80].

## 8. Conclusions

**Figure 3:**
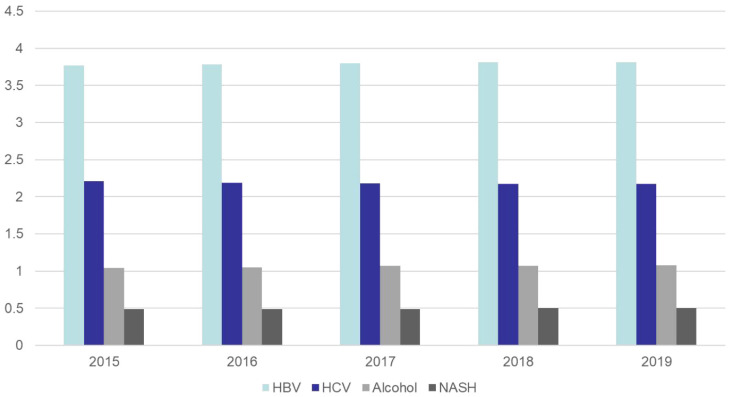
According to the Global Burden of Disease Database, changes in the incidence of liver cancer by different causes in Asia from 2015 to 2019, expressed as rate per 100,000 [5].

East Asia remains one of the regions with the highest burden of hepatocellular carcinoma worldwide [84]. We review that the following factors are contributing to the changing trends in liver cancer in the Asia-Pacific region: the development of country-specific prevention strategies, such as the promotion of healthier lifestyles, the reduce of smoking and alcohol use and community-based health promotion programs; environmental interventions, such as improved grain storage and crop substitution to prevent aflatoxin contamination; better medical interventions, such as new antiviral treatments for patients with HBV or HCV infection (Table 2 and Figure 3) [85].

**Figure 4:**
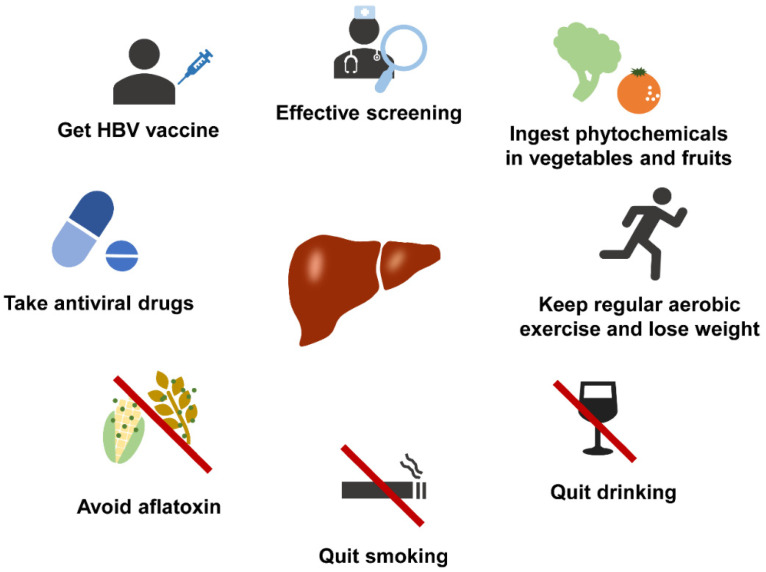
Methods to reduce the incidence of HCC.

With the effective implementation of HBV and treatment regimens for HCV, the epidemiological attribution of HCC is shifting from viral hepatitis to NAFLD [84]. Here, we reviewed the epidemiological trends of HCC in Asia and highlight future efforts, such as improving the lifestyle of patients (Figure 4). Focusing on prevention may be the most effective way to improve the prognosis of this hard-to-treat cancer.

The current effective measures for the prevention and treatment of HCC include vaccination, effective screening, application of antiviral drugs, intake of fresh fruits and vegetables, regular aerobic exercise, weight loss, alcohol prohibition, smoking ban, and avoidance of aflatoxin intake.

## Figures and Tables

**Table 1:** Estimated number of new cases of liver cancer in Asia from 2020 to 2040, Incidence, both sexes, age [0–85+], based on CANCERTOMORROW|IARC.

Population	Annual Population		Number of New Cases		Change in Number of Cases
	2020	2040	2020	2040	
Afghanistan	38,928,341	56,912,008	956	2024	+111.7%
Armenia	2,963,234	2,905,147	427	599	+40.3%
Azerbaijan	10,139,175	11,055,063	510	914	+79.2%
Bahrain	1,701,583	2,199,706	36	109	+202.8%
Bangladesh	164,689,383	188,416,728	3261	5928	+81.8%
Bhutan	771,612	885,158	34	61	+79.4%
Brunei Darussalam	437,483	488,817	43	108	+151.2%
Cambodia	16,718,971	20,526,539	3142	5999	+90.9%
China	1,447,470,092	1,457,962,454	410,038	595,242	+45.2%
Democratic People Republic of Korea	25,778,815	26,858,031	5607	7882	+40.6%
Georgia	3,989,175	3,689,311	418	463	+10.8%
India	1,380,004,391	1,592,691,531	34,743	56,309	+62.1%
Indonesia	273,523,621	318,637,860	21,392	39,273	+83.6%
Iraq	40,222,503	60,583,717	713	1604	+125.0%
Islamic Republic of Iran	83,992,953	98,593,610	5701	12,581	+120.7%
Israel	8,655,541	11,332,909	388	640	+64.9%
Japan	126,476,458	113,356,481	45,663	52,218	+14.4%
Jordan	10,203,140	11,886,728	204	430	+110.8%
Kazakhstan	18,776,707	22,370,403	1039	1707	+64.3%
Kuwait	4,270,563	5,152,526	128	482	+276.6%
Kyrgyzstan	6,524,191	8,307,138	476	911	+91.4%
Lao People Democratic Republic	7,275,556	8,971,941	1272	2453	+92.8%
Lebanon	6,825,442	6,376,397	172	302	+75.6%
Malaysia	32,365,998	38,754,576	2149	3906	+81.8%
Maldives	540,542	556,282	29	83	+186.2%
Mongolia	3,278,292	4,089,199	2236	4506	+101.5%
Myanmar	54,409,794	61,201,610	5466	8529	+56.0%
Nepal	29,136,808	34,889,298	524	1007	+92.2%
Oman	5,106,622	64,37,413	128	364	+184.4%
Pakistan	220,892,331	302,129,186	5331	9901	+85.7%
Palestine	5,101,416	7,599,231	164	407	+148.2%
Philippines	109,581,085	135,618,864	10,594	19,882	+87.7%
Qatar	2,881,060	3,628,689	56	256	+357.1%
Republic of Korea	51,269,183	49,783,741	14,788	23,763	+60.7%
Saudi Arabia	34,813,867	42,473,029	1145	3542	+209.3%
Singapore	5,850,343	6,445,489	1347	3213	+138.5%
Sri Lanka	21,413,250	22,186,243	354	540	+52.5%
Syrian Arab Republic	17,500,657	30,153,278	376	1093	+190.7%
Tajikistan	9,537,642	13,845,878	304	676	+122.4%
Thailand	69,799,978	69,008,295	27,394	42,614	+55.6%
Timor-Leste	1,318,442	1,809,281	48	83	+72.9%
Turkey	84,339,067	94,131,585	5649	10,733	+90.0%
Turkmenistan	6,031,187	7,408,523	317	554	+74.8%
United Arab Emirates	9,890,400	10,648,314	83	452	+444.6%
Uzbekistan	33,469,199	40,608,381	1629	3223	+97.9%
Viet Nam	97,338,583	107,795,035	26,418	41,124	+55.7%
Yemen	29,825,968	42,670,023	745	1541	+106.8%
Totals	4,616,030,644	5,164,031,646	643,637	970,231	+50.7%

**Table 2:** The changing epidemiology of HCC in Asia.

Regions	Main Factor	Trends of HCC
Singapore	HBV	Male incidence decreased from 27.4 cases per 100,000 population in 1973–1977 to 17.2 cases in 2008–2012; Female incidence decreased from 6.9 cases per 100,000 population in 1973–1977 to 4.8.
Taiwan	HBV	The mortality rate decreased from 0·81 deaths per 100,000 to 0·05 per 100,000.
Hong Kong	HBV	The incidence has declined over the past 25 years.
Japan	HCV	The incidence and death have increased exponentially since 1970 and peaked in the early 2000s. After a plateau in 2002–2004, the number of deaths began to decline, reaching 28,889 in 2015.
India	HBV	The incidence has increased over the past two decades.
China	NAFLD	The incidence increased from 3.8% in 2001−2005 to 12.2% in 2006–2010.
Korea	NAFLD	The incidence increased from 3.8% in 2001−2005 to 12.2% in 2006–2010
Philippines	Aflatoxin	The incidence of HCC was reduced.
Qidong	Aflatoxin	A significant decrease in the incidence of HCC in men (ASR = 89.9 from 1983 to 1987, ASR = 60.9 from 2008 to 2012, −32.3%) and a slight decrease in women (ASR = 24.5 from 1983 to 1987, ASR = 21.5 from 2008 to 2012, −12.2%) were observed.
Asia-Pacific region	Alcohol	The increase in alcohol intake across the Asia-Pacific region between 2006 and 2016 May have contributed to an increase in age-standardized liver cancer rates.

## Abbreviations

AAPC	average annual percent change
ASR	Age-Standardized Rate
CHB	chronic hepatitis B
DAA	direct-acting antiviral
HBV	hepatitis B virus
HCC	hepatocellular carcinoma
HCV	hepatitis C virus
IFN	interferon
NAFLD	non-alcoholic liver disease
NAs	nucleos(c)ide analogs
